# Serum CA125 and HE4 as Biomarkers for the Detection of Endometrial Cancer and Associated High-Risk Features

**DOI:** 10.3390/diagnostics12112834

**Published:** 2022-11-17

**Authors:** Chloe E. Barr, Kelechi Njoku, Eleanor R. Jones, Emma J. Crosbie

**Affiliations:** 1 Manchester Academic Health Science Centre, Division of Gynaecology, Manchester NHS Foundation Trust, Manchester M13 9WL, UK; 2 Division of Cancer Sciences, Faculty of Biology, Medicine and Health, University of Manchester, Manchester M13 9PL, UK

**Keywords:** endometrial cancer, diagnosis, HE4, CA125, imaging, non-invasive, biomarkers

## Abstract

Early detection of endometrial cancer improves survival. Non-invasive diagnostic biomarkers would improve triage of symptomatic women for investigations. This study aimed to determine the diagnostic accuracy of serum Cancer Antigen 125 (CA125) and Human Epididymis 4 (HE4) for endometrial cancer and associated high-risk features. Serum samples from women investigated for gynaecological symptoms or diagnosed with endometrial cancer were analysed for CA125 and HE4. Conventional diagnostic metrics were calculated. In total, 755 women were included; 397 had endometrial cancer. Serum CA125 and HE4 were significantly elevated in cases compared with controls (both *p* < 0.001), and with pathological markers of disease severity (*p* < 0.05). A combination of CA125 and HE4 detected endometrial cancer with an area under the curve (AUC) of 0.77 (95% CI: 0.74–0.81). In a model with body mass index (BMI) and parity, HE4 predicted endometrial cancer in pre-menopausal women with an AUC of 0.91 [sensitivity = 84.5%, specificity = 80.9% (*p* < 0.001)]. In women with abnormal ultrasound, HE4 ≥ 77 pmol/L improved specificity compared with imaging alone [68.6% (95% CI: 75.0–83.6) vs. 34.4% (95% CI: 27.1–42.3), respectively], but at a cost to sensitivity. HE4 ≥ 77 pmol/L improved the detection of myometrial invasion ≥50% in women with stage I disease compared with magnetic resonance imaging (MRI) alone [sensitivity = 100% (95% CI: 54.1–100)]. CA125 ≥ 35 U/mL did not add to imaging. HE4 is a good predictor of poor prognostic features which could assist staging investigations.

## 1. Introduction

Endometrial cancer is the commonest gynaecological malignancy in the UK, with an estimated 9500 women diagnosed annually [1]. Overall, it has a good 5-year survival rate of 84% [2] because three-quarters of women are diagnosed at an early, curable stage of disease. However, for those diagnosed at an advanced stage, 5-year survival is poor due to limited effective treatment options. Improving early detection rates is essential to improving outcomes and quality of life for those diagnosed. Around 90% of women present with abnormal uterine bleeding, the majority of which is postmenopausal. However, those who experience less common symptoms, such as vaginal discharge, or are pre-menopausal, can experience diagnostic delays, due to lack of recognition of symptom significance and misattribution to benign causes [3].

In the UK, symptomatic women undergo a transvaginal ultrasound, following which, those with a thickened (≥4 mm) or irregular endometrium have an endometrial biopsy +/− hysteroscopic assessment of the endometrial cavity [4]. Transvaginal ultrasound has an excellent sensitivity [5]; however, it is limited by poor specificity due to benign pathologies such as fibroids, leading to a significant number of women requiring endometrial sampling who ultimately do not have cancer. Whilst endometrial biopsy provides definitive histological diagnosis, the procedure itself is often painful, poorly tolerated and anxiety provoking for patients.

The mainstay of treatment for endometrial cancer is a total hysterectomy and bilateral salpingo-oophorectomy. For a number of women, surgical management is not appropriate, either due to wishes for future fertility or unacceptable surgical risk in those with co-morbidities or significant obesity. The clinical decision regarding conservative management and the extent of surgical intervention is based on pre-operative prognostic indicators including presumed stage on imaging, grade and histological subtype. However, deep myometrial invasion (MI ≥ 50%), cervical stromal invasion and microscopic lymph node metastasis may be difficult to identify on MRI [6], and studies have suggested between 22% and 33% of those with presumed stage IA disease are upstaged on final histology [7,8].

There are no serum biomarkers in routine use for endometrial cancer diagnosis and staging. Cancer antigen 125 (CA125) is a glycoprotein that is in routine use for the diagnosis and monitoring of epithelial ovarian cancer. Human epididymis 4 (HE4) is a whey acidic protein that has been shown to be elevated in a number of cancers including lung [9], ovarian [10], transitional renal cell carcinoma [11] and gastric [12]. Both have shown promise as diagnostic markers for endometrial cancer, and are associated with important markers of disease severity [13]. An accurate diagnostic biomarker could reduce the number of women referred for unnecessary painful and costly investigations, identify those more challenging to diagnose and improve the accuracy of pre-operative staging to aid clinical decisions.

The aim of this study was to determine the accuracy of serum CA125 and HE4 for the detection of endometrial cancer and associated high-risk features.

## 2. Materials and Methods

### 2.1. Study Population

Women attending Manchester University NHS Foundation Trust (MFT) for investigation of gynaecological symptoms or management of endometrial cancer were eligible for inclusion. Women were identified from a database of historical and ongoing endometrial cancer research studies, and had given informed consent for their clinico-pathological data and stored serum samples to be used for future research. Women were excluded if they did not have a pre-treatment serum sample available, if they had recurrent disease at the time of serum sampling or if they had a diagnosis of atypical endometrial hyperplasia.

Women with histologically confirmed endometrial cancer were included as cases. Most women underwent a total hysterectomy +/− a bilateral salpingo-oophorectomy as their primary treatment, with a significant proportion requiring adjuvant therapy in line with national guidance [14]. A number of women with low-grade, early-stage endometrioid tumours underwent conservative management with intrauterine progestins for either fertility sparing reasons, or due to unacceptable surgical risk. Primary radiotherapy was used in a very small number of cases. Histological samples and imaging were reviewed by a consultant histo-pathologist and consultant radiologist, respectively, both with expertise in gynaecological oncology. Where surgery was not the primary treatment, clinical and pathological data were taken from the endometrial biopsy specimen and MRI imaging. The control group included women who had attended the gynaecology department with either symptoms suspicious of endometrial cancer or general gynaecological symptoms. Investigation of postmenopausal bleeding and suspected endometrial cancer was in line with national guidance [14]. Final diagnosis was made based on clinical assessment, imaging and where available, histology. Management of benign conditions was based on individual needs and in line with the relevant guidance.

Women were recruited to contemporaneous studies prospectively, and the database kept up to date. Demographic data collected included age, body mass index (BMI), menopausal status, parity and co-morbidities. Endometrial thickness data were collected from transvaginal ultrasound scan or MRI/CT in cases where no ultrasound was performed. Pathological data included histological subtype, FIGO 2009 stage [15], grade, lymphovascular space invasion (LVSI), depth of MI, cervical stromal invasion and molecular subgroup [16] (if available).

### 2.2. Laboratory Assays

Pre-treatment serum samples were collected with consent by routine venepuncture. Samples were centrifuged at 1500× *g* for 15 min, and then stored in aliquots at −80 °C in the Manchester University NHS Foundation Trust Biobank until testing. Samples were thoroughly thawed to room temperature prior to analysis. Serum HE4 and CA125 were analysed on the Fujirebio Lumipulse^®^ G600II automated analyser, which uses a chemiluminescence enzyme immunoassay (CLEIA) technique. In brief, this is a two-step sandwich immunoassay technique. The luminescence signal produced in the final enzyme reaction is read by the analyser and reflects the amount of analyte in the sample. The Lumipulse^®^ G HE4 immunoreaction cartridges (234068, Fujirebio Europe N.V., Ghent, Belgium) reported limits of detection are 20–1500 pmol/L, and total coefficient of variation (CV) between 3.4% and 5.5%. The limits of detection for Lumipulse^®^ CA125 immunoreaction cartridges (292631, Fujirebio Europe N.V., Gent, Belgium) are 2–1000 U/mL, and the total CV between 2.4–4.0%. Quality controls were run before and after each batch of assays and the protocol for testing was in line with the manufacturer’s instructions.

### 2.3. Statistical Analysis

Continuous data are reported as medians with interquartile ranges as the data were not normally distributed. Non-parametric analysis was performed using a Mann- U- Whitney test or a Kruskal–Wallis test (≥2 or more groups). Categorical data are presented as counts and percentages, and comparison between two groups was performed using Chi-squared analysis. Missing data were completely at random, and so were removed from final analyses.

The performance of serum HE4 and CA125 for the detection of endometrial cancer were analysed using receiver operator characteristics (ROC) curves and the area under the curve (AUC) calculated using the DeLong method with 95% confidence intervals (CI). The sensitivity, specificity, positive predictive value (PPV) and negative predictive value (NPV) of serum biomarkers and transvaginal ultrasound scan (TVS) were calculated using two by two cross tabulations. An abnormal scan was defined as either an ET of ≥4 mm, an irregular endometrium or the presence of a mass in the endometrial cavity [4]. The most commonly used thresholds for HE4 and CA125 in the literature are 70 pmol/L and 35 U/mL, respectively, and are based on a manual enzyme immunoassay (EIA method) [17]. The CLEIA method overestimates HE4 concentrations compared with an EIA; therefore, a cut-off of 77 pmol/L was used to make results comparable [18]. Optimal biomarker thresholds were also explored from the ROC curve using the point-closest-to-(0,1) corner approach [19]. Univariable and multivariable logistic regression models were used to predict the probability of endometrial cancer based on continuous biomarker data and ET. Multivariable models were adjusted for known confounding variables including age, BMI and parity. Models were constructed in a stepwise fashion and the comparison of models was performed using Akaike Information Criterion and likelihood ratio test.

Univariable and multivariable logistic regression models were used to predict the probability of MI ≥ 50%, LVSI and cervical stromal invasion on the final hysterectomy specimen based on continuous biomarker data. ROC curves were constructed based on the univariable models. A subgroup analysis was conducted to assess the accuracy of MRI imaging and serum markers for the detection of MI ≥ 50% in those with FIGO stage I disease. MRI imaging was included in the univariable and multivariable analysis as a categorical variable, and was categorised as positive or negative based on the imaging report. Multivariable models were adjusted for known confounders including stage and grade. The sensitivity, specificity, PPV and NPV were calculated for each marker alone and in combination using the aforementioned thresholds.

A *p*-value of <0.05 indicated significance. Data analyses were performed using STATA (StataCorp. 2015. Stata Statistical Software: Release 14. College Station, TX, USA: StataCorp LLC).

## 3. Results

### 3.1. Study Population Characteristics

**Figure 1:**
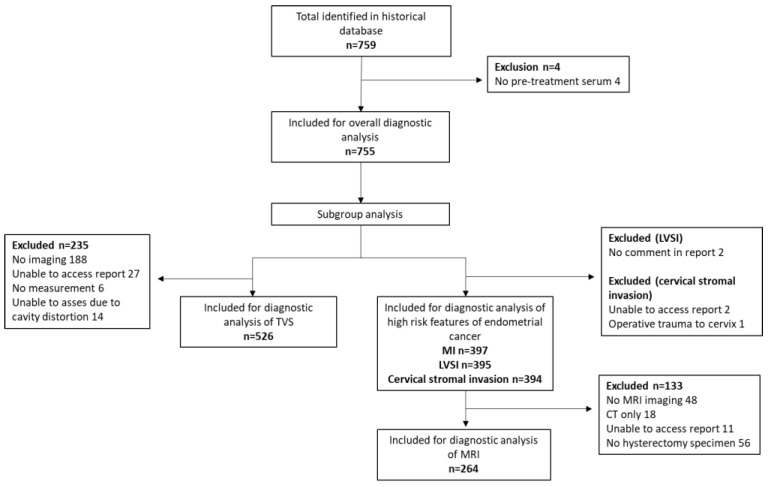
Study flow diagram.

A total of 755 women were eligible for inclusion, of whom 397 (53%) had endometrial cancer (Figure 1).

Their median age and BMI was 64 years (IQR: 53–73) and 31 kg/m^2^ (IQR: 26–40), respectively (Table 1). Endometrial cancer cases were significantly older (66 vs. 58 years, *p* < 0.001) with a higher BMI (32 vs. 30 kg/m^2^, *p* = 0.007) than controls. Most endometrial cancers were FIGO stage I (76%) low grade (70%) endometrioid (80%) tumours (Table 2). Molecular classification was available for 219 (55%) cases and included 10 (5%) *POLE*- mutant, 67 (30%) mismatch repair deficient (MMR-D), 24 (11%) p53 abnormal (p53abn) and 118 (54%) no specific molecular profile (NSMP). The control group included 358 women attending for investigation of gynaecological symptoms (Table 3).

### 3.2. Descriptive Summary of Serum Biomarkers

The median serum CA125 and HE4 of the whole group was 14.9 U/mL (IQR: 10.2–24.7 U/mL) and 93.5 pmol/L (IQR: 66.0–144.2 pmol/L), respectively. Table 4 summarises the serum markers in relation to clinical and pathological characteristics of the participants. Serum levels of HE4 (123.9 pmol/L vs. 73.6 pmol/L, *p* < 0.001) and CA125 (18.8 U/mL vs. 11.8 U/mL, *p* < 0.001) were found to be significantly higher in women with endometrial cancer compared with those without. Both serum HE4 and CA125 were significantly higher in those with advanced FIGO stage (*p* = 0.02 and *p* < 0.001), high grade (*p* = 0.01 and *p* = 0.006), MI ≥ 50% (both *p* < 0.001) and LVSI (both *p* < 0.001). No association was observed between the serum markers and histological subtype or molecular classification. HE4 and CA125 levels were significantly correlated (Spearman’s Rho 0.46, *p* < 0.001). HE4 was significantly correlated with age (Spearman’s Rho 0.52, *p* < 0.001), whereas CA125 demonstrated only a weak correlation (Spearman’s Rho 0.14, *p* = <0.001). There was no association between either biomarker and BMI (CA125: Spearman’s Rho 0.03, *p* = 0.36. HE4: Spearman’s Rho −0.06, *p* = 0.11).

### 3.3. CA125 and HE4 as Diagnostic Biomarkers for Endometrial Cancer

In the total study population, serum HE4 was a better diagnostic biomarker for endometrial cancer than CA125 (AUC 0.76 vs. 0.71, respectively, *p* = 0.03) (Figure S1). When adjusted for age, BMI, menopausal status and parity in the multivariable model, the combination of HE4 and CA125 predicted endometrial cancer with an AUC of 0.79 (sensitivity 67%, specificity 78%).

Using the literature thresholds of 77 pmol/L and 35 U/mL for HE4 and CA125, respectively, HE4 was more sensitive [79.3% (95% CI: 75.0–83.2) vs. 24.9% (95% CI: 20.8–29.5)], but less specific [53.1% (95% CI: 47.8–58.3) vs. 94.7% (95% CI: 91.8–96.8)] than CA125 (Table 5). HE4 and CA125, where either was positive, showed the highest sensitivity for endometrial cancer (80.2%, 95% CI: 76.4–84.4). The diagnostic performance of optimal thresholds from the ROC curve analysis is shown in Table S1.

HE4 and CA125 were less accurate for the detection of endometrial cancer in pre-menopausal compared with postmenopausal women [HE4: AUC 0.75 (95% CI: 0.65–0.84) vs. 0.78 (95% CI: 0.74–0.81), CA125: AUC 0.67 (95% CI: 0.56–0.77) vs. 0.72 (95% CI: 0.68–0.76)] (Figure S2). However, after adjustment for BMI and parity in the multivariable analysis the model performance of HE4 improved in pre-menopausal women (AUC 0.91, sensitivity 84.5%, specificity 80.9%, *p* < 0.001). The performance of HE4 and/or CA125 at pre-specified and data-driven thresholds according to menopausal status is shown in Table 5 and Supplementary Table S1.

### 3.4. CA125 and HE4 as Triage Biomarkers for Intrauterine Investigations in Women with Abnormal Transvaginal Ultrasound Scan Findings

Of the 526 women who had scan results available, 462 (88%) had abnormal scan findings of which 357 had endometrial cancer [sensitivity 97.5% (95% CI: 95.4–98.9), specificity 34.4% (95% CI: 27.1–42.3)]. Adding HE4 ≥ 77 pmol/L to determine which women with abnormal scan findings underwent invasive investigations improved specificity [68.6% (95% CI: 75.0–83.6)] but at a cost to sensitivity [79.6% (95% CI: 58.8–77.3)]. CA125 ≥ 35 U/mL demonstrated a particularly poor sensitivity [24.6% (95% CI: 20.3–29.5)] and would not be clinically useful at this threshold (Table 6).

In a model that included ET and HE4 as continuous variables (*n* = 426), the AUC was 0.89, sensitivity 86.8% and specificity 76.3% (Figure S3). Adding in age and BMI improved the sensitivity (sensitivity 89.0%, specificity 72.3% and AUC 0.89) but CA125 and menopausal status did not significantly add to diagnostic performance (Table S2).

### 3.5. CA125 and HE4 as Biomarkers of High-Risk Endometrial Cancer

Serum CA125 and HE4 levels were significantly elevated in the presence of pathological features of high-risk disease, including high grade, advanced stage, MI ≥ 50% and LVSI (Table 4).

**Figure 2:**
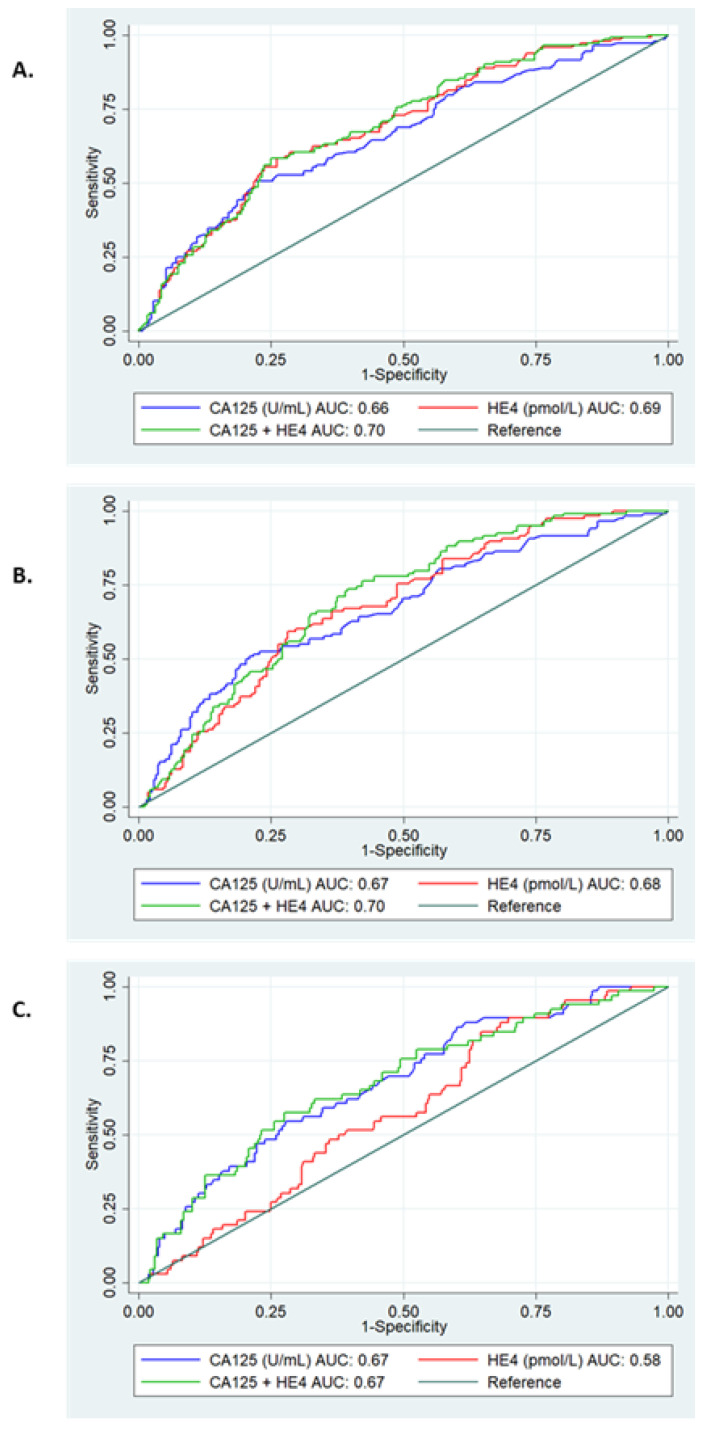
ROC curve analysis of serum markers for the detection of high-risk features of endometrial cancer. (**A**)—Myometrial invasion. CA125 AUC 0.66 (95% CI 0.61–0.72), HE4 AUC 0.69 (95% CI 0.64–0.74), combined AUC 0.70 (95% CI 0.64–0.75), *p* = 0.01. (**B**)—LVSI. CA125 AUC 0.67 (95% CI 0.61–0.73), HE4 AUC 0.68 (95% CI 0.62–0.73), combined AUC 0.70 (95% CI 0.65–0.76), *p* = 0.006. (**C**)—Cervical stromal invasion. CA125 AUC 0.67 (95% CI 0.60–0.74), HE4 AUC 0.58 (95% CI 0.51–0.64), combined AUC 0.67 (95% CI 0.60–0.74), *p* = 0.02.

Figure 2A shows the diagnostic performance of the markers for the detection of MI ≥ 50%. Overall, HE4 demonstrated the best performance with an AUC of 0.69 (95% CI: 0.64–0.74). The addition of CA125 to HE4 did not significantly improve diagnostic performance [AUC 0.70 (95% CI: 0.64–0.75), *p* = 0.67). Adjusting for age, BMI, stage and grade improved the diagnostic performance of HE4, with an AUC of 0.78 (Table S3).

For the detection of LVSI, HE4 was superior to CA125 [AUC 0.68 (95% CI: 0.62–0.73) vs. AUC 0.67 (95% CI: 0.61–0.73)] and the combination of the two markers did not improve the performance of HE4 alone (*p* = 0.28) (Figure 2B). When adjusted for confounding variables, the AUC improved to 0.79 (Table S3).

CA125 performed better than HE4 for the detection of cervical stromal invasion with an AUC of 0.67 (95% CI: 0.60–0.74) (Figure 2C). Overall, as continuous data, neither HE4 nor CA125 were significant predictors of cervical stromal invasion in the univariable analysis (*p* = 0.68 and *p* = 0.15, respectively) (Table S3).

### 3.6. Diagnostic Accuracy of Serum Biomarkers and MRI for the Detection of Deep MI

Of those who had MRI imaging, 188 were FIGO stage I on final histology and 53 (28%) had MI ≥ 50%. MRI was able to detect MI ≥ 50% with a sensitivity of 88.7% (95% CI: 77.0–95.7) and a specificity of 67.4% (95% CI: 58.8–75.2) (Table 7). The addition of HE4 ≥ 77 pmol/L in those with a normal MRI had a sensitivity of 100% (95% CI: 54.1–100), detecting all of those with MI ≥ 50% that were missed on MRI; however, the specificity was 33.0% (95% CI: 23.5–43.6).

In a model that included both MRI and HE4 (as a continuous variable), the AUC was 0.83, the sensitivity 50.9% and specificity 87.4% (Table S4). Adjustment for histological subtype and grade improved overall model performance (AUC 0.84) and specificity (91.1%) but at a cost to sensitivity (47.1%). CA125 as a continuous variable was not a significant predictor of MI ≥ 50% in those with stage I disease, and did not add to the diagnostic accuracy of the combination of MRI and HE4.

## 4. Discussion

### 4.1. Main Findings

In this study, we explored the diagnostic accuracy of serum CA125 and HE4 for the detection of endometrial cancer. CA125 and HE4 distinguished endometrial cancer cases from healthy and symptomatic controls, with HE4 demonstrating potential utility as a biomarker in pre-menopausal women, in whom diagnosis is more challenging. The addition of HE4 to TVS to identify those requiring further invasive testing improved specificity but at a cost to sensitivity. CA125 and HE4 were found to be significantly elevated in the presence of high-risk pathological characteristics, and HE4 ≥ 77 pmol/L was able to detect all cases of MI ≥ 50% in those with stage I disease, in whom MRI was falsely negative. HE4 more than CA125 shows potential utility for the detection of endometrial cancer, and if our findings were confirmed in larger prospective studies, may have a role in screening, triaging women for invasive investigation and aiding pre-operative planning.

### 4.2. Strengths and Limitations

To the best of our knowledge, this is one of the largest studies investigating the diagnostic accuracy of CA125 and HE4 in endometrial cancer. Our study benefits from a control group that includes women with abnormal bleeding and gynaecological symptoms, which reflects the population in whom the biomarkers would be used. This is an important consideration as the diagnostic performance of biomarkers is influenced by different populations, known as the spectrum effect [20]. Furthermore, we had information on diagnostic and pre- operative imaging for half to two-thirds of our population, allowing direct comparison of the diagnostic performance of the serum biomarkers to current techniques and how they might add to current pathways.

Whilst the participants were recruited to historical studies prospectively, we recognise the limitation of the retrospective nature of this study. As a result, we are limited by missing imaging data. Furthermore, our study is a single centre study based in the North-West of England, and so may not be reflective of other populations and treatment centres. Our centre does not routinely undertake lymphadenectomy for early-stage disease, due to low risk of metastases and limited evidence of benefit [4]; therefore, we have been unable to explore the potential utility of the serum markers for the detection of lymph node metastases. Small numbers limited the extent of the analysis in pre-menopausal women, and further work would be required to assess the utility of HE4 and CA125 in symptomatic pre-menopausal women and whether the markers could improve the accuracy of endometrial thickness in this cohort. Whilst our results are promising for the use of serum CA125 and HE4 as diagnostic biomarkers for endometrial cancer and prediction of high-risk features, we recognise that serum biomarkers will not replace imaging and definitive histopathology. Serum biomarkers may have a role in endometrial cancer screening, improving endometrial cancer detection in pre-menopausal women, and aid pre-operative clinical decision making in combination with MRI. Further larger studies are warranted to establish the true clinical benefit of these serum biomarkers in the endometrial cancer diagnostic pathway.

### 4.3. Comparison with the Existing Literature

Serum CA125 and HE4 have been extensively studied as diagnostic biomarkers for ovarian cancer, and there has been growing interest in their utility in endometrial cancer over the last few years. Several small studies have shown that serum CA125 and HE4 levels are elevated in women with endometrial cancer compared with those without, and have reported on their potential clinical utility [21,22,23,24,25,26,27,28,29,30]. We found the diagnostic accuracy of HE4 to be superior to CA125 (*p* = 0.03), which is supported by much of the literature; however, in contrast to other studies, we only observed a small difference in diagnostic performance between HE4 (AUC 0.76) and CA125 (AUC 0.71). Whilst the performance of HE4 in our study is comparable to that of a large meta-analysis by Chen et al. (pooled AUC 0.77), the authors report a significantly poorer AUC for CA125 (pooled AUC 0.37) [31]. Similarly, a meta-analysis by Li et al., which included 1106 endometrial cancer cases and 1480 controls, reported an AUC of 0.58 for CA125 and an AUC of 0.88 for HE4 [17]. Few studies have evaluated the combination of HE4 and CA125 for endometrial cancer diagnosis. Those that have, report the combination of the biomarkers has a superior performance to either marker alone, with AUC’s ranging from 0.78 to 0.90 [21,24,25,30,32].

No studies have evaluated the serum markers in combination with endometrial thickness for endometrial cancer detection; however, urine CA125 and HE4 in combination with ET has been investigated, with a reported improvement in AUC with the addition of urine CA125 to ET compared with ET alone (AUC 0.97 vs. 0.94, respectively) [33].

It is well documented that HE4 and CA125 are associated with histo-pathological markers of disease severity, and several studies have evaluated the utility of these biomarkers to detect MI ≥ 50%, LVSI and cervical stromal invasion. We found that HE4 and CA125 are able to detect MI ≥ 50% (AUC 0.69 and 0.66, respectively) with moderate accuracy, and this is supported by the literature [7,34,35,36]; however, neither marker was significantly predictive of cervical stromal invasion. A large prospective study by Antonsen et al. reported similar AUCs to our study for both biomarkers for the detection of MI ≥ 50%; however, when dichotomised at the same thresholds, HE4 had a significantly poorer sensitivity (59.8% vs. 96.2%) than our study, despite similar populations [34]. Few studies have evaluated biomarker performance in comparison with pre-operative MRI for predicting high-risk features within the same study population [36,37]. In 68 women with endometrial cancer, Zamani et al. reported that in those with MI ≥ 50%, 40.0% had a CA125 ≥ 35 U/mL, 37.9% an HE4 ≥ 140 pmol/L, and 68.9% had a positive MRI [36]. Whilst our findings are similar in relation to CA125 ≥ 35 U/mL, we found that MRI and HE4 had much higher sensitivities than those suggested by Zamani et al. This is likely due to differences in population size, the threshold used for HE4 and expertise of MRI reporting.

### 4.4. Clinical and Research Implications

Early detection of endometrial cancer improves survival and the quality of life of those affected; however, despite early diagnosis in a large proportion of women, around a quarter of women present with aggressive or advanced stage disease, with limited effective treatment options and poor outcomes. The majority of women with endometrial cancer present with postmenopausal bleeding, leading to the development of the National Institute of Health and Care Excellence (NICE) ‘Suspected Cancer: Recognition and Referral’ clinical care guideline (NG12), which sets out a list of criteria that trigger referral for endometrial cancer investigations [38]. However, age over 55 years and postmenopausal bleeding are the only criteria in which referral is recommended, and referral of other symptoms ‘to be considered’. Postmenopausal bleeding is an extremely common presentation to cancer exclusion clinics, but only around 5% of those will have a diagnosis of endometrial cancer, leading to unnecessary pain, discomfort and anxiety for 95% of women, with associated cost implications to the healthcare system. Furthermore, urgent referral of women outside the aforementioned criteria is less clear. Other symptoms indicative of endometrial cancer include irregular, heavy or intermenstrual bleeding, abnormal vaginal discharge, abdominal pain, urinary symptoms and haematuria and occasionally bowel symptoms [3]. Often, women with these symptoms, in particular those who are pre-menopausal, experience delays in diagnosis which are attributable to a lack of recognition of the significance of symptoms and treatment of presumed benign causes [39]. Furthermore, diagnosis using TVS in pre-menopausal women is challenging, as the ET fluctuates cyclically due to hormonal influences.

No screening test exists for endometrial cancer in either the general or high-risk populations. Women with Lynch syndrome, an inherited disorder affecting one of the four mismatch repair genes (*MLH1*, *MSH2*, *MSH6* and *PMS2*), have an increased lifetime risk of endometrial cancer of up to 50%, as well as a number of other malignancies [3]. Whilst prophylactic total hysterectomy +/− bilateral salpingo-oophorectomy is recommended to reduce lifetime risk, TVS and endometrial biopsy are often used as screening tools for those in whom surgery is unacceptable. However, there is limited evidence that these painful and invasive tests improve outcomes [4].

There is an urgent need for diagnostic biomarkers that could be used as screening tools and for triaging symptomatic women for further invasive investigation for endometrial cancer, whilst safely reassuring those with a negative test. Our study suggests that HE4 and, to a lesser extent CA125, show promise as non-invasive endometrial cancer diagnostic biomarkers, discriminating endometrial cancer cases from healthy and symptomatic controls with a combined AUC of 0.77, and a sensitivity of 80.6% at published thresholds, using the strategy where either marker was positive. Furthermore, HE4 may be of benefit for endometrial cancer detection in pre-menopausal women, with our study showing a model incorporating HE4, BMI and parity could detect endometrial cancer with an AUC of 0.91. Several factors influence serum CA125 in pre-menopausal women, including menstrual cycle fluctuations and benign gynaecological disease, making it less useful as a biomarker in younger women, something which has also been shown in ovarian cancer [40]. Whilst serum HE4 is less influenced by these factors, making it potentially more useful than CA125 in pre-menopausal women, its association with age complicates interpretation in postmenopausal women [41,42]. Combining HE4 and CA125 in models with menopausal status improves ovarian cancer detection compared with either marker alone [43,44], including for pre-menopausal women [45]. Research is needed to determine whether a similar such model could be developed and validated for use for endometrial cancer. Further work is required to determine how these markers might perform in women with Lynch syndrome, and to establish their utility alongside ultrasound scan in pre-menopausal women.

The majority of women with endometrial cancer are diagnosed with low grade, early-stage disease and undergo surgical management, including a total hysterectomy and bilateral salpingo-oophorectomy. Despite inclusion of lymph node status in the FIGO staging of endometrial cancer, the role of routine lymphadenectomy remains unclear, due to increased surgical complexity, patient morbidity and limited clinical benefit in early-stage low grade disease. For some women, surgical management is not an option due to their desire to retain fertility or unacceptable surgical risk due to co-morbidities. Decisions regarding the extent of surgical resection and appropriateness of conservative management rely largely on pre-operative imaging and staging. However, microscopic nodal metastasis, cervical stromal involvement and MI ≥ 50% are challenging to identify on MRI alone, and several studies have reported 22–33% of women with stage IA disease were upstaged on final histology with 33% diagnosed with MI ≥ 50% and 8.2% diagnosed with pelvic nodal involvement in those with grade 1 disease [7,8]. We found that serum HE4 was associated with poor prognostic pathological features, and identified all those with MI ≥ 50% with normal MRI imaging, suggesting HE4 may assist in pre-treatment staging. It is known that MI ≥ 50%, LVSI and grade are associated with risk of lymph node metastasis [46], and whilst we were not able to assess the value of HE4 and CA125 in detection of lymph node metastasis due to small numbers of women undergoing lymphadenectomy at our unit, we have shown the utility of HE4 and CA125 to detect these associated high-risk features, which may indicate lymphadenectomy may be appropriate in those with a raised HE4. Indeed, several studies have demonstrated the promising utility of HE4 in detecting lymph node involvement [34,47,48,49,50,51,52], suggesting HE4 may be useful in aiding risk stratification of women for lymphadenectomy.

Blood tests are simple, relatively non-invasive and cheap to perform, and serum HE4 and CA125 may have a role in screening and detection of those at high risk of endometrial cancer, and those in whom diagnosis is more challenging. These features are also important to patients and clinicians as highlighted in the James Lind Alliance Priority Setting Partnership for Detecting Cancer Early, where ‘what simple, non-invasive, painless, cost-effective and convenient diagnostic tests can be used to detect cancer early’ was ranked first of the top ten priorities for early cancer detection [53].

Despite the promising evidence for the utility of HE4 and CA125 in endometrial cancer detection and management, there is still much work to be carried out before implementing these markers in routine clinical care. Many of the studies are limited by their retrospective design, small sample sizes and differences in control populations. However, by far the biggest barrier to clinical use is the lack of consensus in optimal biomarker thresholds. The most common thresholds used for both HE4 and CA125 are those used for ovarian cancer detection [54,55]; however, the clinical and molecular profile of endometrial cancer differs to that of ovarian cancer, and so these thresholds are unlikely to be clinically useful. In our study, we found the optimal threshold of HE4 and CA125 to be 99 pmol/L and 15 U/mL, respectively. Several other studies have suggested similar optimal thresholds of 113 pmol/L for HE4 and 20 U/mL for CA125 [7,34,56]. Furthermore, whilst we have demonstrated that the sensitivity of current imaging improves with the addition of serum HE4 at a threshold of 77 pmol/L, this comes at a cost to specificity, which in practice would lead to increased numbers referred for investigation and/or more invasive management with associated patient anxiety, morbidity and cost to the healthcare service. Further research is required to optimise and validate endometrial cancer specific thresholds and associated healthcare costs. It is likely that unique thresholds would be required for different aspects of the patient journey from diagnosis to management.

## 5. Conclusions

There remains a need for accurate biomarkers for endometrial cancer screening, detection and to assist decisions around management. In this study we have shown that serum HE4 more than CA125 is a promising diagnostic biomarker, and is associated with histo-pathological markers of disease severity that may aid in pre-operative staging. However, larger prospective studies are needed to confirm these findings and optimise endometrial cancer specific thresholds.

## Figures and Tables

**Table 1:** Characteristics of study population.

	All Participants *n* = 755	No EC *n* = 358	EC *n* = 397	*p* Value (No EC vs. EC)
**Age (years)**				
Median (IQR)	64 (53–73)	58 (52–72)	66 (57–73)	<0.001
**BMI (kg/m^2^)**				
Median (IQR)	31 (26–40)	30 (25–40)	32 (27–41)	0.007
**Menopausal**				
No Yes Missing data	108 (14) 643 (85) 4 (1)	50 (14) 305 (85) 3 (1)	58 (14) 338 (85) 1 (1)	0.83
**Parity**				
Nulliparous Multiparous Missing data	127 (17) 582 (77) 46 (6)	44 (12) 287 (80) 27 (8)	83 (21) 295 (74) 19 (5)	0.003
**T2DM**				
No Yes Missing	630 (83) 123 (16) 2 (1)	303 (84.6) 54 (15) 1 (0.4)	327 (82) 69 (17) 1 (1)	0.394
**Hypertension**				
No Yes Missing	455 (60) 298 (39) 2 (1)	219 (61.1) 138 (38.5) 1 (0.4)	236 (59) 160 (40) 1 (1)	0.624

EC—endometrial cancer. *n*—number. IQR—interquartile range. BMI—body mass index. T2DM—type 2 diabetes mellitus.

**Table 2:** Pathological characteristics of endometrial cancers *n* = 397.

	Number (%)
**Histological subtype**	
Endometrioid Clear Cell Serous Mucinous Carcinosarcoma Mixed	317 (80) 15 (4) 27 (6) 2 (0.5) 28 (7.5) 8 (2)
**Grade**	
1 2 3	190 (48) 88 (22) 119 (30)
**FIGO 2009 Stage**	
I II III IV	302 (76) 40 (10) 45 (11) 10 (3)
**Molecular classification (*n* = 219, 55%)**	
*POLE*-M MMR-D p53abn NSMP	10 (5) 67 (30) 24 (11) 118 (54)
**LVSI (*n* = 395, 99%)**	
No Yes	277 (70) 118 (30)
**Myometrial Invasion**	
No Yes	253 (64) 144 (36)
**Cervical Stromal Invasion (*n* = 394, 99%)**	
No Yes	328 (83) 66 (17)

LVSI—lymphovascular space invasion. *n*—number.

**Table 3:** Final diagnosis of control group.

Benign Pathology	Number (%)
Normal endometrium Atrophy Endometrial polyp Cervical polyp Endometritis Fibroid	267 (75%) 48 (13%) 33 (9%) 3 (0.8%) 1 (0.2%) 6 (2%)

**Table 4:** Summary of serum CA125 and HE4 and clinico-pathological characteristics.

Variable	Median CA125, U/mL (IQR)	*p*-Value	Median HE4, pmol/L (IQR)	*p*-Value
**a** **Clinical**				
**Age (years)**				
<65 ≥65	13.9 (9.9–20.8) 16.3 (10.7–28.3)	0.001	71.4 (55.0–105.0) 121.2 (90.9–196.5)	<0.001
**BMI (kg/m^2^)**				
<30 ≥30	14.2 (9.8–22) 15.4 (10.5–25.9)	0.11	94.7 (68.9–139.7) 93.0 (62.4–156.2)	0.53
**Menopausal**				
No Yes	14.3 (10.0–22.8) 15.0 (10.2–25.1)	0.26	63.8 (50.5–86.95) 99 (69.7–156.5)	<0.001
**T2DM**				
No Yes	15.0 (10.4–24.8) 14.7 (9.6–24.2)	0.35	92.6 (65.9–141.7) 98.9 (66.0–162.1)	0.47
**b** **Pathological**				
**Histological diagnosis**				
Benign EC	11.8 (9.1–17.2) 18.8 (12.6–34.6)	<0.001	73.6 (56.2–98.0) 123.9 (81.8–198.4)	<0.001
**Stage**				
I+II III+IV	18.2 (12.1–30.1) 34.6 (17.7–77.2)	<0.001	121.3 (79.7–186.1) 163.8 (96.1–298.3)	0.02
**Grade**				
1+2 3	18.4 (12.1–31.1) 20.7(14.5–43.5)	0.006	118.8 (76.9–183.7) 134.0 (99.0–228.0)	**0.01**
**Myometrial Invasion**				
<50% ≥50%	17.3 (11.7–26.6) 27.6 (15.8–53.01)	<0.001	108.2 (75.1–158.0) 165.0 (104.4–281.8)	<0.001
**LVSI**				
Absent Present	17.4 (11.7–27.0) 30.2 916.2–55.1)	<0.001	109.1 (75.8–162.8) 207.2 (12.5–401.8)	<0.001
**Histological Subtype**				
EEC Non-EEC	18.8 (12.5–33.6) 19.8 (13.4–43.5)	0.19	122.3 (79.7–189.5) 131.6 (93.3–228.0)	0.19
**Cervical Stromal invasion**				
No Yes	18.2 (12.2–30.4) 29.8 (17.2–65.1)	<0.001	121.2 (77.9–195.1) 141.9 (99.1–196.5)	0.05
**Molecular Classification**				
*POLE*-M MMR-D NSMP p53Abn	22.1 (18.2–45.1) 18.4 (13.1–31.9) 18.25 (11.5–32.0) 16.6 (12.0–36.2)	0.61	139.9 (77.1–189.5) 141.1 (99.1–246.6) 121.0 (82.7–183.6) 162.05 (97.9–230.0)	0.33

EC—endometrial cancer. *n*—number. IQR—interquartile range. BMI—body mass index. T2DM—type 2 diabetes mellitus.

**Table 5:** Diagnostic accuracy of serum CA125 and HE4 for the detection of endometrial cancer.

Biomarker	Histology		Total	Diagnostic Accuracy			
	No EC, *n*	EC, *n*		Sensitivity, % (95% CI)	Specificity, % (95% CI)	PPV, % (95% CI)	NPV, % (95% CI)
**a** **Total cohort** (*n* = 755, EC 397 (53%))							
**CA125 (U/mL)**							
<35	339	298	637				
≥35	19	99	118	24.9 (20.8–29.5)	94.7 (91.8–96.8)	83.9 (76.0–90.0)	53.2 (49.3–57.1)
**HE4 (pmol/L)**							
<77 pmol/L	190	82	272				
≥77 pmol/L	168	315	483	79.3 (75.0–83.2)	53.1 (47.8–58.3)	65.2 (60.8–69.5)	69.9 (64.0–75.2)
**Combined**							
Negative	186	77	263				
Positive *	172	320	492	80.6 (76.4–84.4)	52.0 (46.6–57.2)	65.0 (60.6–69.3)	70.7 (64.8–76.2)
**b** **Pre-menopausal women** (*n* = 108, EC= 58 (54%))							
**CA125 (U/mL)**							
<35	50	51	101				
≥35	0	7	7	12.1 (4.99–23.3)	100 (92.9–100)	100 (59–100)	49.5 (39.4–59.6)
**HE4 (pmol/L)**							
<77 pmol/L	41	9	50				
≥77 pmol/L	9	30	58	51.7 (38.2–65.0)	82.0 (68.6–91.4)	76.9 (60.7–88.9)	59.4 (46.9–71.1)
**Combined**							
Negative	41	28	69				
Positive *	9	30	39	51.7 (38.2–65.0)	82.0 (68.6–91.4)	76.9 (60.7–88.9)	59.4 (46.9–71.1)
**c** **Postmenopausal women** (*n* = 643, EC= 338 (53%))							
**CA125 (U/mL)**							
<35	287	246	533				
≥35	18	92	110	27.2 (22.5–32.3)	94.1 (90.8–96.5)	83.6 (75.4–90.0)	53.8 (49.5–58.1)
**HE4 (pmol/L)**							
<77 pmol/L	148	53	201				
≥77 pmol/L	157	285	442	84.3 (80.0–88.0)	48.5 (42.8–54.3)	64.5 (59.8–68.9)	73.6 (67.0–79.6)
**Combined**							
Negative	144	48	192				
Positive *	161	290	451	85.8 (81.6–89.3)	47.2 (41.5–53.0)	64.3 (59.7–68.7)	75.0 (68.3–81.0)

* either positive. CI—confidence interval. PPV—positive predictive value. NPV—negative predictive value. *n*—number. EC—endometrial cancer.

**Table 6:** Diagnostic accuracy of TVS imaging and serum biomarkers for the detection of endometrial cancer.

Biomarker/ Imaging	Histology		Total	Diagnostic Accuracy			
	No EC, *n*	EC, *n*		Sensitivity, % (95% CI)	Specificity, % (95% CI)	PPV, % (95% CI)	NPV, % (95% CI)
**a** **Total cohort** (*n* = 526, EC= 366 (70%))							
**TVS**							
Normal	55	9	64				
Abnormal	105	357	462	97.5 (95.4–98.9)	34.4 (27.1–42.3)	77.3 (73.2–81.0)	85.9 (75.0–93.4)
**CA125 (U/mL)**							
<35	152	277	429				
≥35	8	89	97	24.3 (20.0–29.0)	95.0 (90.4–97.8)	91.8 (84.4–96.4)	35.4 (30.9–40.2)
**HE4 (pmol/L)**							
<77	105	76	181				
≥77	55	290	345	79.2 (74.7–83.3)	65.6 (57.7–72.9)	84.1 (79.8–87.8)	58.0 (50.5–65.3)
**b** **Abnormal imaging** (*n* = 462, EC = 357 (77%)							
**CA125 (U/mL)**							
<35	100	269	369				
≥35	5	88	93	24.6 (20.3–29.5)	95.2 (89.2–98.4)	94.6 (87.9–98.2)	27.1 (22.6–31.9)
**HE4 (pmol/L)**							
<77 pmol/L	72	73	145				
≥77 pmol/L	33	284	317	79.6 (75.0–83.6)	68.6 (58.8–77.3)	89.6 (85.7–92.7)	49.7 (41.3–58.1)
**Combined**							
Negative	70	68	138				
Positive *	35	289	324	81.0 (76.5–84.9)	66.7 (56.8–75.6)	89.2 (85.3–92.4)	50.7 (42.1–59.3)

* either positive. CI—confidence interval. PPV—positive predictive value. NPV—negative predictive value. TVS—transvaginal ultrasound. EC—endometrial cancer. *n*—number.

**Table 7:** Diagnostic accuracy of MRI imaging and serum markers for the detection of MI ≥ 50% in those with FIGO stage I endometrial cancer.

Biomarker/ Imaging	Histology		Total	Diagnostic Accuracy			
	MI < 50%, *n*	MI ≥ 50%, *n*		Sensitivity, % (95% CI)	Specificity, % (95% CI)	PPV, % (95% CI)	NPV, % (95% CI)
**a** **FIGO 2009 Stage I** (*n* = 188, MI ≥ 50% = 53 (28%))							
**MRI**							
Normal	91	6	97				
Abnormal	44	47	91	88.7 (77.0–95.7)	67.4 (58.8–75.2)	51.6 (40.9–62.3)	93.8 (87.0–97.7)
**CA125 (U/mL)**							
<35	115	33	148				
≥35	20	20	40	37.7 (24.8–52.1)	85.2 (78.1–90.7)	50.0 (33.8–66.2)	77.7 (70.1–84.1)
**HE4 (pmol/L)**							
<77	34	2	36				
≥77	101	51	152	96.2 (87.0–99.5)	25.2 (18.1–33.4)	33.6 (26.1–41.7)	94.4 (81.3–99.3)
**b** **Abnormal imaging** (*n* = 91, MI ≥ 50% = 47 (52%))							
**CA125 (U/mL)**							
<35	35	29	64				
≥35	9	18	27	38.3 (24.5–53.6)	79.5 (64.7–90.2)	66.7 (46.0–83.5)	54.7 (41.7–67.2)
**HE4 (pmol/L)**							
<77 pmol/L	4	2	6				
≥77 pmol/L	40	45	85	95.7 (85.5–99.5)	9.09 (2.53–21.7)	52.9 (41.8–63.9)	66.7 (22.3–95.7)
**Combined**							
Negative	4	1	5				
Positive *	40	46	86	97.9 (88.7–99.9)	9.09 (2.53–21.7)	53.5 (42.4–64.3)	80.0 (28.4–99.5)
**c** **Normal imaging** (*n* = 97, MI ≥ 50%= 6 (6%))							
**CA125 (U/mL)**							
<35	80	4	84				
≥35	11	2	13	33.3 (4.3–77.7)	87.9 (79.4–93.8)	15.4 (1.92–45.4)	95.2 (88.3–98.7)
**HE4 (pmol/L)**							
<77 pmol/L	30	0	30				
≥77 pmol/L	61	6	67	100 (54.1–100)	33.0 (23.5–43.6)	8.96 (3.36–18.5)	100 (88.4–100)
**Combined**							
Negative	28	0	28				
Positive *	63	6	69	100 (54.1–100)	30.8 (21.5–41.3)	8.7 (3.26–18.0)	100 (87.7–100)

* either positive. CI—confidence interval. PPV—positive predictive value. NPV—negative predictive value. MI—myometrial invasion. MRI—magnetic resonance imaging. *n*—number.

## Data Availability

Fully anonymised data are available on reasonable request to the corresponding author.

## Supplementary Materials

The following supporting information can be downloaded at: https://www.mdpi.com/article/10.3390/diagnostics12112834/s1, Figure S1: ROC analysis of serum CA125 and serum HE4 for the detection of endometrial cancer; Table S1: Diagnostic accuracy of CA125 and HE4 for the detection of endometrial cancer at optimal thresholds title; Figure S2: ROC analysis of CA125 and HE4 for the detection of endometrial cancer stratified by menopausal status; Figure S3: ROC curve analysis of serum markers and endometrial thickness models for the detection of endometrial cancer; Table S2: Univariable and multivariable logistic regression models of serum markers and ET for the prediction of endometrial cancer; Table S3: Univariable and multivariable logistic regression models of serum markers for the prediction of high-risk features of endometrial cancer; Table S4: Univariable and multivariable logistic regression models of serum markers and MRI for the prediction of MI ≥ 50% in those with stage I endometrial cancer (n = 188, MI ≥ 50% = 53 (28%)).

## References

[1] Cancer Research UK (CRUK) Uterine Cancer Incidence Statistics 2017. https://www.cancerresearchuk.org/health-professional/cancer-statistics/statistics-by-cancer-type/uterine-cancer/incidence.

[2] Cancer Research UK (CRUK) Uterine Cancer Survival Statistics 2015. https://www.cancerresearchuk.org/health-professional/cancer-statistics/statistics-by-cancer-type/uterine-cancer/survival.

[3] Crosbie E.J., Kitson S.J., McAlpine J.N., Mukhopadhyay A., Powell M.E., Singh N. (2022). Endometrial cancer. Lancet.

[4] Morrison J., Balega J., Buckley L., Clamp A., Crosbie E., Drew Y., Durrant L., Forrest J., Fotopoulou C., Gajjar K. (2022). British Gynaecological Cancer Society (BGCS) uterine cancer guidelines: Recommendations for practice. Eur. J. Obstet. Gynecol. Reprod. Biol..

[5] Timmermans A., Opmeer B.C., Khan K.S., Bachmann L.M., Epstein E., Clark T.J., Gupta J.K., Bakour S., van den Bosch T., van Doorn H.C. (2010). Endometrial thickness measurement for detecting endometrial cancer in women with postmenopausal bleeding: A systematic review and meta-analysis. Obstet. Gynecol..

[6] Sanjuán A., Escaramís G., Ayuso J.R., Román S.M., Torné A., Ordi J., Lejárcegui J.A., Pahisa J. (2008). Role of magnetic resonance imaging and cause of pitfalls in detecting myometrial invasion and cervical involvement in endometrial cancer. Arch. Gynecol. Obstet..

[7] Panyavaranant P., Manchana T. (2020). Preoperative markers for the prediction of high-risk features in endometrial cancer. World J. Clin. Oncol..

[8] Sirisabya N., Manchana T., Worasethsin P., Khemapech N., Lertkhachonsuk R., Sittisomwong T., Vasuratna A., Termrungruanglert W., Tresukosol D. (2009). Is complete surgical staging necessary in clinically early-stage endometrial carcinoma?. Int. J. Gynecol. Cancer.

[9] Choi S.I., Jang M.A., Jeon B.R., Shin H.B., Lee Y.K., Lee Y.W. (2017). Clinical usefulness of human epididymis protein 4 in lung cancer. Ann. Lab. Med..

[10] Huang J., Chen J., Huang Q. (2018). Diagnostic value of HE4 in ovarian cancer: A meta-analysis. Eur. J. Obstet. Gynecol. Reprod. Biol..

[11] Xi Z., LinLin M., Ye T. (2009). Human epididymis protein 4 is a biomarker for transitional cell carcinoma in the urinary system. J. Clin. Lab. Anal..

[12] O’Neal R.L., Nam K.T., LaFleur B.J., Barlow B., Nozaki K., Lee H.J., Kin W.H., Yang H.-K., Shi C., Maitra A. (2013). Human epididymis protein 4 is up-regulated in gastric and pancreatic adenocarcinomas. Hum. Pathol..

[13] Behrouzi R., Barr C.E., Crosbie E.J. (2021). HE4 as a Biomarker for endometrial cancer. Cancers.

[14] Sundar S., Balega J., Crosbie E., Drake A., Edmondson R., Fotopoulou C., Gallos I., Ganesan R., Gupta J., Johnson N. (2017). BGCS uterine cancer guidelines: Recommendations for practice. Eur. J. Obstet. Gynecol. Reprod. Biol..

[15] Pecorelli S. (2009). Revised FIGO staging for carcinoma of the vulva, cervix, and endometrium. Int. J. Gynaecol. Obstet..

[16] Masood M., Singh N. (2021). Endometrial carcinoma: Changes to classification (WHO 2020). Diagn. Histopathol..

[17] Li J., Wang X., Qu W., Wang J., Jiang S.W. (2019). Comparison of serum human epididymis protein 4 and CA125 on endometrial cancer detection: A meta-analysis. Clin. Chim. Acta..

[18] Barr C.E., Funston G., Mounce L.T.A., Pemberton P.W., Howe J.D., Crosbie E.J. (2021). Comparison of two immunoassays for the measurement of serum HE4 for ovarian cancer. Pract. Lab. Med..

[19] Liu X. (2012). Classification accuracy and cut point selection. Stat. Med..

[20] Usher-Smith J.A., Sharp S.J., Griffin S.J. (2016). The spectrum effect in tests for risk prediction, screening, and diagnosis. BMJ.

[21] Moore R.G., Brown A.K., Miller M.C., Badgwell D., Lu Z., Allard W.J., Granai C.O., Bast R.C., Lu K. (2008). Utility of a novel serum tumor biomarker HE4 in patients with endometrioid adenocarcinoma of the uterus. Gynecol. Oncol..

[22] Liu J.-L., Gao X.-L., Hou C.-Z., Lin Z., Xu H. (2018). Diagnostic Significance of Changes in Serum Human Epididymis Epithelial Secretory Protein 4 and Carbohydrate Antigen 125 in Endometrial Carcinoma Patients. Acta. Medica. Mediterr..

[23] Abdalla N., Pazura M., Słomka A., Piórkowski R., Sawicki W., Cendrowski K. (2016). The role of HE4 and CA125 in differentiation between malignant and non-malignant endometrial pathologies. Ginekol. Pol..

[24] Zanotti L., Bignotti E., Calza S., Bandiera E., Ruggeri G., Galli C., Tognon G., Ragnoli M., Romani C., Tassi R.A. (2012). Human epididymis protein 4 as a serum marker for diagnosis of endometrial carcinoma and prediction of clinical outcome. Clin. Chem. Lab. Med..

[25] Dong C., Liu P., Li C. (2017). Value of HE4 combined with cancer antigen 125 in the diagnosis of endometrial cancer. Pak. J. Med. Sci..

[26] Dewan R., Dewan A., Hare S., Bhardwaj M., Mehrotra K. (2017). Diagnostic performance of serum human epididymis protein 4 in endometrial carcinoma: A pilot study. JCDR.

[27] Angioli R., Plotti F., Capriglione S., Montera R., Damiani P., Ricciardi R., Aloisi A., Luvero D., Cafà E.V., Dugo N. (2013). The role of novel biomarker HE4 in endometrial cancer: A case control prospective study. Tumor Biol..

[28] Bian J., Sun X., Li B., Ming L. (2016). Clinical significance of serum HE4, CA125, CA724, and CA19–9 in patients with endometrial cancer. Technol. Cancer Res. Treat..

[29] Bignotti E., Ragnoli M., Zanotti L., Calza S., Falchetti M., Lonardi S., Berqamelli S., Bandiera E., Tassi R.A., Romani C. (2011). Diagnostic and prognostic impact of serum HE4 detection in endometrial carcinoma patients. Br. J. Cancer.

[30] Omer B., Genc S., Takmaz O., Dirican A., Kusku-Kiraz Z., Berkman S., Gurdol F. (2013). The diagnostic role of human epididymis protein 4 and serum amyloid-A in early-stage endometrial cancer patients. Tumor Biol..

[31] Chen Y., Ren Y.L., Li N., Yi X.F., Wang H.Y. (2016). Serum human epididymis protein 4 vs. carbohydrate antigen 125 and their combination for endometrial cancer diagnosis: A meta-analysis. Eur. Rev. Med. Pharmacol. Sci..

[32] Huang G.Q., Xi Y.Y., Zhang C.J., Jiang X. (2019). Serum human epididymis protein 4 combined with carbohydrate antigen 125 for endometrial carcinoma diagnosis: A meta-analysis and systematic review. Genet. Test Mol. Biomarkers..

[33] Njoku K., Barr C.E., Sutton C.J.J., Crosbie E.J. (2022). Urine CA125 and HE4 for the triage of symptomatic women with suspected endometrial cancer. Cancers.

[34] Antonsen S.L., Høgdall E., Christensen I.J., Lydolph M., Tabor A., Loft Jakobsen A., Fagö-Olsen C.L., Andersen E.S., Jochumsen K., Høgdall C. (2013). HE4 and CA125 levels in the preoperative assessment of endometrial cancer patients: A prospective multicenter study (ENDOMET). Acta. Obstet. Gynecol. Scand..

[35] Brennan D.J., Hackethal A., Metcalf A.M., Coward J., Ferguson K., Oehler M.K., Quinn M.A., Janda M., Leung Y., Freemantle M. (2014). Serum HE4 as a prognostic marker in endometrial cancer—A population based study. Gynecol Oncol..

[36] Zamani N., Modares Gilani M., Zamani F., Zamani M.H. (2015). Utility of Pelvic MRI and Tumor Markers HE4 and CA125 to Predict Depth of Myometrial Invasion and Cervical Involvement in Endometrial Cancer. J. Fam. Reprod. Health..

[37] Rajadevan N., McNally O., Neesham D., Richards A., Naaman Y. (2021). Prognostic value of serum HE4 level in the management of endometrial cancer: A pilot study. Aust. N. Z. J. Obstet. Gynaecol..

[38] National Institue for Health and Care Excellence (NICE) Suspected Cancer: Recognition and Referral. https://www.nice.org.uk/guidance/ng12.

[39] Zhou Y., Mendonca S.C., Abel G.A., Hamilton W., Walter F.M., Johnson S., Shelton J., Elliss-Brookes L., McPhail S., Lyratzopoulos G. (2018). Variation in ’fast-track’ referrals for suspected cancer by patient characteristic and cancer diagnosis: Evidence from 670,000 patients with cancers of 35 different sites. Br. J. Cancer..

[40] Sandri M.T., Bottari F., Franchi D., Boveri S., Candiani M., Ronzoni S., Peiretti M., Radice D., Passerini R., Sideri M. (2013). Comparison of HE4, CA125 and ROMA algorithm in women with a pelvic mass: Correlation with pathological outcome. Gynecol. Oncol..

[41] Moore R.G., Miller M.C., Steinhoff M.M., Skates S.J., Lu K.H., Lambert-Messerlian G., Bast R.C. (2012). Serum HE4 levels are less frequently elevated than CA125 in women with benign gynecologic disorders. Am. J. Obstet. Gynecol..

[42] Moore R.G., Miller M.C., Eklund E.E., Lu K.H., Bast R.C., Lambert-Messerlian G. (2012). Serum levels of the ovarian cancer biomarker HE4 are decreased in pregnancy and increase with age. AJOG.

[43] Wilailak S., Chan K.K.L., Chen C.-A., Nam J.-H., Ochiai K., Aw T.-C., Sabaratnam S., Hebbar S., Sickan J., Schodin B.A. (2015). Distinguishing benign from malignant pelvic mass utilizing an algorithm with HE4, menopausal status, and ultrasound findings. J. Gynecol. Oncol..

[44] Moore R.G., McMeekin D.S., Brown A.K., DiSilvestro P., Miller M.C., Allard W.J., Gajewski W., Kurman R., Bast R.C., Skates S.J. (2009). A novel multiple marker bioassay utilizing HE4 and CA125 for the prediction of ovarian cancer in patients with a pelvic mass. Gynecol. Oncol..

[45] Barr C.E., Funston G., Jeevan D., Sundar S., Mounce L.T.A., Crosbie E.J. (2022). The Performance of HE4 Alone and in Combination with CA125 for the Detection of Ovarian Cancer in an Enriched Primary Care Population. Cancers.

[46] Stålberg K., Kjølhede P., Bjurberg M., Borgfeldt C., Dahm-Kähler P., Falconer H., Holmberg E., Staf C., Tholander B., Åvall-Lundqvist E. (2017). Risk factors for lymph node metastases in women with endometrial cancer: A population-based, nation-wide register study—On behalf of the Swedish Gynecological Cancer Group. Int. J. Cancer.

[47] Abbink K., Zusterzeel P.L., Geurts-Moespot A.J., Herwaarden A.E.V., Pijnenborg J.M., Sweep F.C., Massuger L.F. (2018). HE4 is superior to CA125 in the detection of recurrent disease in high-risk endometrial cancer patients. Tumour Biol..

[48] Dobrzycka B., Mackowiak-Matejczyk B., Terlikowska K.M., Kinalski M., Terlikowski S.J. (2016). Utility of HE4 to identify patients with endometrioid endometrial cancer who may require lymphadenectomy. Adv. Med. Sci..

[49] Gao M., Gao Y. (2021). Value of preoperative neutrophil–lymphocyte ratio and human epididymis protein 4 in predicting lymph node metastasis in endometrial cancer patients. J. Obstet. Gynaecol. Res..

[50] Gąsiorowska E., Magnowska M., Iżycka N., Warchoł W., Nowak-Markwitz E. (2016). The role of HE4 in differentiating benign and malignant endometrial pathology. Ginekol. Pol..

[51] O’Toole S.A., Huang Y., Norris L., Power Foley M., Shireen R., McDonald S., Kamran W., Ibrahim N., Ward M., Thompson C. (2021). HE4 and CA125 as preoperative risk stratifiers for lymph node metastasis in endometrioid carcinoma of the endometrium: A retrospective study in a cohort with histological proof of lymph node status. Gynecol. Oncol..

[52] Wang Y., Han C., Teng F., Bai Z., Tian W., Xue F. (2017). Predictive value of serum HE4 and CA125 concentrations for lymphatic metastasis of endometrial cancer. IJGO.

[53] Badrick E., Cresswell K., Ellis P., Renehan A.G., Crosbie E.J., Detecting Cancer Early Priority Setting Partnership Steering Group (appendix) (2019). Top ten research priorities for detecting cancer early. Lancet Public Health.

[54] National Institute for Health and Care Excellence (NICE) Ovarian Cancer: Recognition and Initial Management Clinical Guideline 122 (CG122). https://www.nice.org.uk/Guidance/CG1222011.

[55] Moore R.G., Brown A.K., Miller M.C., Skates S., Allard W.J., Verch T., Steinhoff M., Messerlian G., DiSilvestro P., Granai C.O. (2008). The use of multiple novel tumor biomarkers for the detection of ovarian carcinoma in patients with a pelvic mass. Gynecol. Oncol..

[56] Kurihara T., Mizunuma H., Obara M., Andoh K., Ibuki Y., Nishimura T. (1998). Determination of a normal level of serum CA125 in postmenopausal women as a tool for preoperative evaluation and postoperative surveillance of endometrial carcinoma. Gynecol. Oncol..

