# Supplementary material for: Serum CA125 and HE4 as Biomarkers for the Detection of Endometrial Cancer and Associated High-Risk Features
Source: Diagnostics (Basel). 2022 Nov 17;12(11):2834. doi: 10.3390/diagnostics12112834 (PMC9689358; doi:10.3390/diagnostics12112834)
Supplement: Supplementary file 1 [file diagnostics-12-02834-s001.zip › diagnostics-1997368-supplementary.pdf]

Serum CA125 and HE4 as Diagnostic Biomarkers in Endometrial Cancer- Supplementary Materials

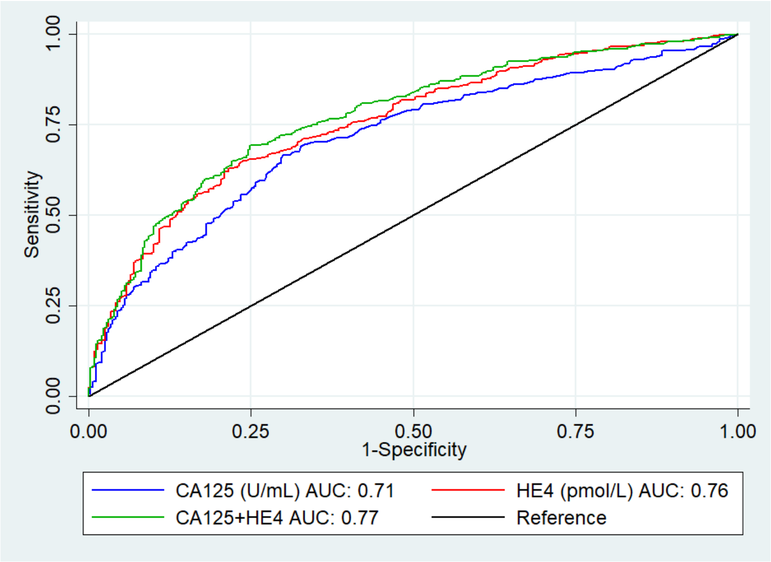

Figure S1. ROC analysis of serum CA125 and serum HE4 for the detection of endometrial cancer. n=755. CA125 AUC 0.71 (95%CI 0.68-0.75). HE4 AUC 0.76 (95%CI 0.72-0.79). Combined AUC 0.77 (95%CI 0.74-0.81).

Table S1. Diagnostic accuracy of CA125 and HE4 for the detection of endometrial cancer at **optimal** thresholds.

	**Sensitivity % (95%CI)**	**Specificity % (95%CI)**	**PPV (95%CI)**	**NPV (95%CI)**
**a. Total cohort (n=755, EC 397 (53%))**				
CA125 (≥15U/mL)	66.8 (61.9-71.4)	69 (63.9-73.8)	70.5 (65.6-75.0)	65.2 (60.1-70.0)
HE4 (≥99pmol/L)	65.2 (60.3-69.9)	75.7 (70.9-80.1)	74.9 (69.9-79.3)	66.3 (61.4-70.8)
Combined (either positive)	82.6 (78.5-86.2)	57.3 (52.0-62.4)	68.2 (63.8- 72.3)	74.8 (69.2-79.8)
**b. Pre-menopausal** *(n=108 EC=58 (54%))*				
CA125 (≥15U/mL)	62.1 (48.4-74.5)	70 (55.4-82.1)	70.6 (56.2-82.5)	61.4 (47.6-74)
HE4 (≥99pmol/L)	34.5 (22.5-48.1)	94 (83.5-98.7)	87 (66.4-97.2)	55.3 (44.1-66.1)
Combined (either positive)	70.7 (57.3-81.9)	66 (51.2-78.8)	70.7 (57.3-81.9)	66 (51.2-78.8)
**c. Postmenopausal** *(n= 643 EC= 338 (53%))*				
CA125 (≥15U/mL)	67.5 (62.2-72.4)	69.2 (63.7-74.3)	70.8 (65.5-75.7)	65.7 (60.3-70.9)
HE4 (≥99pmol/L)	70.7 (65.5-75.5)	72.8 (67.4-77.7)	74.2 (69.1-78.9)	69.2 (63.8-74.2)

Combined (either positive)	84.6 (80.3-88.3)	56.1 (50.3-61.7)	68.1 (63.4-72.5)	76.7 (70.6-82.1)

CI- confidence interval. PPV- positive predictive value. NPV- negative predictive value. n-number. EC-endometrial cancer.

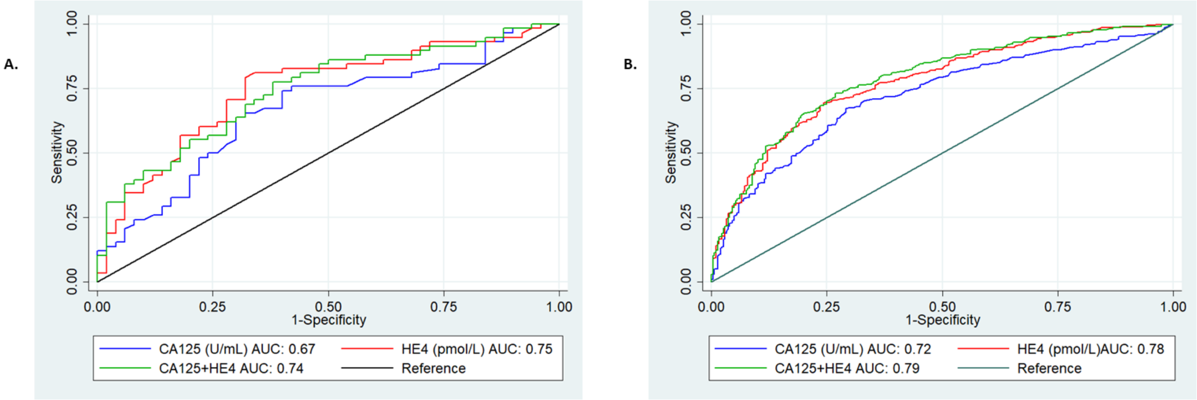

Figure S2. ROC analysis of CA125 and HE4 for the detection of endometrial cancer stratified by menopausal status**. A- Pre menopausal.** CA125 AUC 0.67 (95%CI 0.56-0.77), HE4 AUC 0.75 (0.65-0.84), Combined AUC 0.74 (95%CI 0.65-0.84). **B- Postmenopausal.** CA125 AUC 0.72 (95%CI 0.68-0.76), HE4 AUC 0.78 (95%CI 0.74-0.81), Combined AUC 0.79 (95%CI 0.75-0.82).

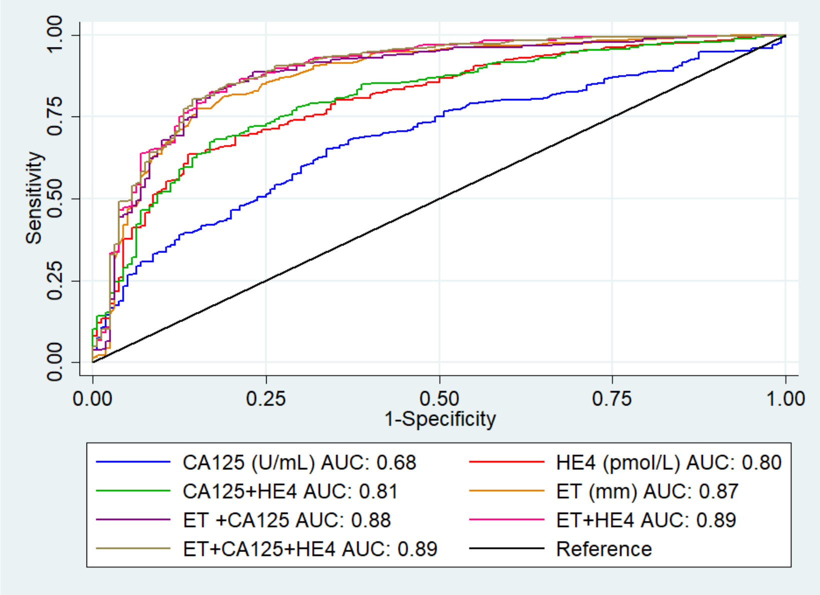

Figure S3. ROC curve analysis of serum markers and endometrial thickness models for the detection of endometrial cancer. CA125 AUC 0.68 (95%CI 0.64-0.73). HE4 AUC 0.80 (95%CI 0.76-0.84). CA125 + HE4 AUC 0.81 (95%CI 0.77-0.85). ET AUC 0.87 (95%CI 0.84-0.91). ET+CA125 AUC 0.88 (0.84-0.91). ET+HE4 AUC 0.89 (95% 0.86-0.92). ET+HE4+CA125 AUC 0.89 (95%CI 0.86-0.92).

Table S2. Univariable and multivariable logistic regression models of serum markers and ET for the prediction of endometrial cancer (n=426, EC= 326 (77%)).

Model	Predictor	Univariable		Multivariable				
		Crude OR (95%CI)	p-value	Adjusted OR (95%CI)	p-value	AUC	Sens	Spec
**A. Base model**								
**ET**	Disease status	1.21 (1.16-1.26)	<0.001	1.20 (1.15-1.25)	**<0.001**	0.88	89.9	70.4
**B. Base model + HE4**								
**HE4**	Disease status	1.02 (1.01-1.03)	<0.001	1.01 (1.00-1.02)	**0.001**	0.89	89.0	72.3
**ET**				1.17 (1.12-1.23)	**<0.001**			
**C. Base model + HE4 + CA125**								
**CA125**	Disease status	1.04 (1.02-1.06)	<0.001	1.01 (0.99-1.03)	0.29	0.89	89.3	72.3
**HE4**				1.01 (1.00-1.01)	**0.002**			
**ET**				1.17 (1.12-1.22)	**<0.001**			

OR- odds ratio. CI- confidence interval. AUC- area under the curve. Multivariable analysis adjusted for age and BMI. Akaike Information Criterion (AIC) model A-433.50. AIC model B- 417.91. AIC model C- 418.43. Comparison of models (likelihood ratio test): A vs B p<0.001. B vs C p=0.22.

Table S3. Univariable and multivariable logistic regression models of serum markers for the prediction of high risk features of endometrial cancer.

		Univariable Analysis		Multivariable Analysis				
**Model**	**Predictor**	**Crude OR (95%CI)**	**p-value**	**Adjusted OR (95%CI)**	**p-value**	**AUC**	**Sens**	**Spec**
**HE4**	Myometrial Invasion	1.002 (1.001-1.003)	<0.001	1.002 (1.000-1.003)	0.001	0.78	43.1%	88.9%

**CA125**		1.003 (0.999-1.007)	0.06					
**HE4**	LVSI	1.002 (1.000-1.003)	0.004	1.002 (1.000-1.003)	0.003	0.79	41.5%	92.8%
**CA125**		1.003 (0.999-1.006)	0.07					
**HE4**	Cervical stromal invasion	1.00 (0.999-1.001)	0.68					
**CA125**		1.002 (0.999-1.005)	0.154	1.000 (0.997-1.004)	0.81	0.77	10.6%	97.0%

OR- odds ratio. CI- confidence interval. AUC- area under the curve. **Myometrial invasion:** Multivariable analysis adjusted for age, BMI, stage and grade (model AIC 430.01), histological subtype (AIC 430.83, LRT p=0.28) and CA125 (AIC 431.69, LRT p=0.57) added no benefit to model. **LVSI:** Multivariable analysis adjusted for age, BMI, stage, grade and histological subtype (model AIC 396.13), CA125 added no benefit to model (AIC 398.11, LRT p=0.88). **Cervical stromal invasion:** Multivariable adjusted for stage and histological subtype (AIC 320.07), grade (AIC 319.90, LRT 0.14) added no benefit to model. CA125 added no benefit to base model of confounders (AIC 320.07 vs 318.13, LRT p=0.81).

Table S4. Univariable and multivariable logistic regression models of serum markers and MRI for the prediction of MI≥50% in those with stage I endometrial cancer (n=188, MI≥50%= 53 (28%)).

**Model**	**Predictor**	**Univariable**		**Multivariable**				
		**Crude OR (95%CI)**	**p-value**	**Adjusted OR (95%CI)**	**p-value**	**AUC**	**Sens**	**Spec**
**A. Base model**								
**MRI**	MI≥50%	16.2 (6.44-40.76)	**<0.001**	17.9 (6.88-46.77)	**<0.001**	0.81	32.1	92.6
**B. Base model + HE4**								
**HE4**	MI≥50%	1.002 (1.000-1.003	**0.03**	1.002 (1.000-1.004)	**0.04**	0.84	47.2	91.1
**MRI**				18.0 (6.77-47.9)	**<0.001**			
**C. Base model + HE4 + CA125**								

**CA125**	MI≥50%	1.001 (0.997-1.004)	0.63	0.997 (0.993-1.001)	0.29	0.84	47.2	88.2
**HE4**				1.003 (1.000-1.005)	**0.03**			
**MRI**				19.0 (7.00-51.37)	**<0.001**			

OR- odds ratio. CI- confidence interval. AUC- area under the curve. Multivariable analysis adjusted for histological subtype and grade. AIC model A-176.46. AIC model B- 174.21. AIC model C- 175.09. Comparison of models (likelihood ratio test): A vs B p=0.04. B vs C p=0.29.
